# *Trypanosoma brucei* pteridine reductase 1 is essential for survival *in vitro* and for virulence in mice

**DOI:** 10.1111/j.1365-2958.2010.07236.x

**Published:** 2010-06-15

**Authors:** Natasha Sienkiewicz, Han B Ong, Alan H Fairlamb

**Affiliations:** Division of Biological Chemistry & Drug Discovery, College of Life Sciences, University of DundeeDundee, UK

## Abstract

Gene knockout and knockdown methods were used to examine essentiality of pteridine reductase (PTR1) in pterin metabolism in the African trypanosome. Attempts to generate PTR1 null mutants in bloodstream form *Trypanosoma brucei* proved unsuccessful; despite integration of drug selectable markers at the target locus, the gene for PTR1 was either retained at the same locus or elsewhere in the genome. However, RNA interference (RNAi) resulted in complete knockdown of endogenous protein after 48 h, followed by cell death after 4 days. This lethal phenotype was reversed by expression of enzymatically active *Leishmania major* PTR1 in RNAi lines (^oe^RNAi) or by addition of tetrahydrobiopterin to cultures. Loss of PTR1 was associated with gross morphological changes due to a defect in cytokinesis, resulting in cells with multiple nuclei and kinetoplasts, as well as multiple detached flagella. Electron microscopy also revealed increased numbers of glycosomes, while immunofluorescence microscopy showed increased and more diffuse staining for glycosomal matrix enzymes, indicative of mis-localisation to the cytosol. Mis-localisation was confirmed by digitonin fractionation experiments. RNAi cell lines were markedly less virulent than wild-type parasites in mice and virulence was restored in the ^oe^RNAi line. Thus, PTR1 may be a drug target for human African trypanosomiasis.

## Introduction

Tetrahydrobiopterin (H_4_B) is an essential cofactor for various hydroxylation reactions catalysed by enzymes such as aromatic amino acid hydroxylases, glyceryl ether monooxygenases and NO synthases (Thony *et al*., 2000; Werner-Felmayer *et al*., 2002; Schmidt and Alp, 2007; Watschinger *et al*., 2009). In humans, H_4_B can be synthesized *de novo* from GTP or salvaged from dihydrobiopterin (H_2_B) via NADPH-dependent dihydrofolate reductase (DHFR, EC 1.5.13) (Nichol *et al*., 1983) or regenerated from quinonoid dihydrobiopterin, a by-product of hydroxylation reactions, via NAD(P)H-dependent 6,7-dihydropteridine reductase (DHPR; EC 1.5.1.34) (Armarego *et al*., 1983).

In contrast to humans, biological and genomic data indicate that trypanosomatids lack the ability to synthesize pterins *de novo*, and are totally dependent on salvage of extracellular pterins for growth (Bello *et al*., 1994; Cunningham and Beverley, 2001; Berriman *et al*., 2005; Ivens *et al*., 2005). The essential requirement for pterins was initially discovered in *Crithidia fasciculata* grown in a defined medium containing low amounts of folate (Kidder and Dutta, 1958; Kaufman, 1963); and our subsequent understanding of the uptake, salvage and functions of pterins in trypanosomatids has come principally from studies by the Beverley and Ouellette groups (Nare *et al*., 1997; Ouellette *et al*., 2002). In *Leishmania spp.*, biopterin is taken up from the medium via the biopterin transporter BT1 (Kundig *et al*., 1999; Lemley *et al*., 1999) and then sequentially reduced to H_2_B and H_4_B by pteridine reductase 1 (PTR1; EC 1.5.1.33) (Bello *et al*., 1994). Previous studies on *Leishmania ptr1*^-^ mutants suggested the presence of a residual pteridine reductase activity, provisionally assigned as PTR2. This activity is considered not to be due to quinonoid dihydropteridine reductase and the identity of PTR2 remains unknown (Lye *et al*., 2002). Some kinetic, inhibitor and structural properties have been reported for PTR1 from *Leishmania major* (Luba *et al*., 1998; Gourley *et al*., 2001; Schuttelkopf *et al*., 2005), *Trypanosoma cruzi* (Schormann *et al*., 2005) and *Trypanosoma brucei* (Dawson *et al*., 2006; Mpamhanga *et al*., 2009; Shanks *et al*., 2010). Gene knockout studies in *L. major* have demonstrated that PTR1 is essential for growth of the insect promastigote stage of the parasite, where growth of *ptr1*^-^ null mutants could be restored with H_2_B or H_4_B, but not by dihydro- or tetrahydrofolate (Bello *et al*., 1994). These mutants showed increased formation of mammalian-infective metacyclic promastigote forms in stationary phase cultures and consequently gave larger lesions when injected into mice (Cunningham *et al*., 2001). This indicates that *ptr1*^-^ amastigotes are viable *in vivo*, presumably due to abundant H_4_B in host macrophages and consequently that PTR1 is not a drug target on its own in *L. major*.

PTR1 has a secondary role in metabolism in being able to reduce dihydrofolate to tetrahydrofolate. Amplification of PTR1 in *Leishmania spp.* is one of the several mechanisms by which parasites acquire resistance to antifolates such as methotrexate (Callahan and Beverley, 1992; Papadopoulou *et al*., 1992; Bello *et al*., 1994) either by acting as a by-pass mechanism or by sequestering these DHFR inhibitors. Recent studies have demonstrated an intriguing link between unconjugated pterins and oxidative susceptibility in a variety of *Leishmania* PTR1 knockout and overexpressing cell lines (Moreira *et al*., 2009; Nare *et al*., 2009). However, the mechanism involved is not clear; neither is it known whether pterins have additional essential functions in these parasites.

In contrast to *Leishmania spp.* virtually nothing is known about pterin metabolism in African trypanosomes, parasites that occupy a completely different (extracellular) environment in the mammalian host. In this study we use genetic methods to examine the role of PTR1 in blood stream form *T. brucei* with respect to essentiality *in vitro* and infectivity *in vivo*. Our findings suggest that reduced pterins are required for glycosome and flagellar biogenesis and that loss of PTR1 activity cannot be compensated for by reduced pterins in plasma and extracellular fluids. Thus, unlike *L. major*, PTR1 is a potential drug target in the African trypanosome without reference to DHFR.

## Results

### Genotypic and phenotypic analysis of TbPTR1 knockdown in parasites

To assess the essentiality of *PTR1*, classical gene replacement methods were undertaken to try to produce a *PTR1* null cell line of bloodstream trypanosomes. Consistent with genome sequence data for strain 927 (Berriman *et al*., 2005), Southern blot analysis confirmed that *PTR1* is single copy per haploid genome in the ‘single marker’ bloodstream *T. brucei* 427 used in these studies (data not shown). This organism, subsequently referred to as wild-type (WT), constitutively expresses T7 RNA polymerase and the tetracycline repressor protein, and can be used to express other RNA constructs under the control of tetracycline (Wirtz *et al*., 1999). Replacement of one copy of PTR1 to generate a single knockout (SKO) could be readily achieved by transfection with a construct containing a drug resistance gene (either HYG or PAC) flanked by the 5′- and 3′-untranslated regions of *PTR1*, followed by drug selection with either hygromycin or puromycin, essentially as described (Sienkiewicz *et al*., 2008). SKO lines (*PTR1/ptr1::HYG*[SKO*^HYG^*] or *PTR1/ptr1::PAC*[SKO*^PAC^*]) were then subjected to a second round of transfection with the other knockout construct and selected for resistance to both drugs. Despite obtaining double drug-resistant trypanosomes in numerous independent experiments, we were unable to obtain clones in which both copies of *PTR1* had been deleted. Southern blotting confirmed that integration of the drug-resistant genes had indeed occurred at the correct locus, but was associated with retention of an additional copy of PTR1 either at the same locus or elsewhere in the genome (see Fig. S1). In virulent strains of leishmania, such behaviour is typical of an essential gene (Cruz *et al*., 1993), but has not been reported for the African trypanosome. However, an increase in chromosomal copy number has been reported for trypanosomes selected for resistance to mycophenolic acid, an inhibitor of IMP dehydrogenase (Wilson *et al*., 1994). Attempts to generate conditional double knockout cell lines by introducing an ectopic copy of PTR1 (pLew100_*TbPTR1*) under tetracycline control were also unsuccessful.

**Fig. 1:**
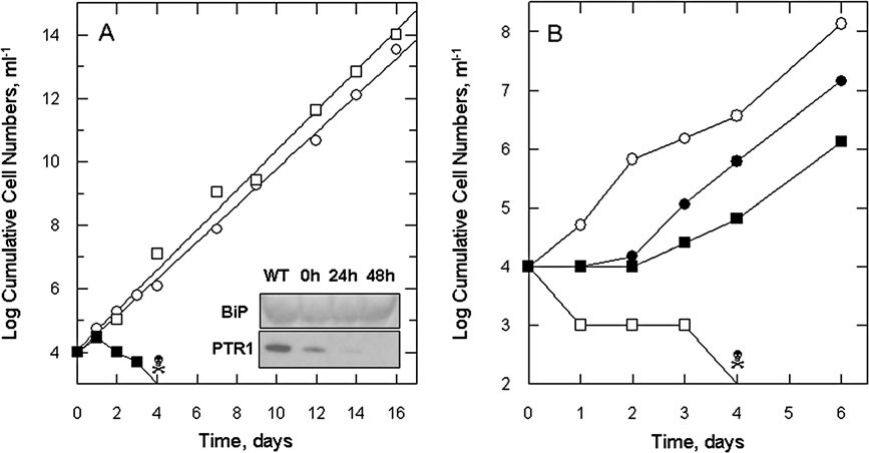
The effect of RNAi-dependent depletion of PTR1 on growth in the presence or absence of tetrahydrobiopterin. A. Effect of RNAi on cell growth. Four independent RNAi clones were analysed for growth defects following induction with tetracycline in the absence of tetrahydrobiopterin. WT (open circles) and RNAi cells (non-induced cells – open squares and induced cells – closed squares) were seeded at 1 × 10^4^ cells ml^−1^ and growth was determined over a 2 week period. WT, non-induced and induced cells (1 × 10^6^ cells per lane) were harvested at 0 (non-induced), 24 h and 48 h (induced) and processed for Western blot analysis. The blot was sequentially probed with anti-*Tb*PTR1 (inset, lower panel) and anti-*Tb*BIP (inset, upper panel) as loading control. B. Effect of tetrahydrobiopterin on cell growth. WT cells and a freshly derived RNAi clone were seeded into FDM or HMI9-T supplemented with or without 10 µM tetrahydrobiopterin at 1 × 10^4^ cells ml^−1^ and growth monitored over 1 week. Symbols: WT (open circle; no additions), non-induced RNAi cells (closed circles) and tetracycline-induced cells plus or minus tetrahydrobiopterin (closed and open squares respectively). Data shown for FDM only; HMI9-T shows essentially the same effect.

In the light of these results, inducible RNA interference (RNAi) studies using the p2T7^Ti^TAblue system were undertaken to deplete cells of PTR1 (Alibu *et al*., 2005). For the disruption of PTR1 function, a 403 bp region was ligated into the p2T7^Ti^TAblue vector and a linearized construct transfected into WT cells by electroporation. Cells that had integrated the construct into the rDNA loci by homologous recombination were obtained by selection with hygromycin and then cloned. Generation of the correct RNAi clones was confirmed by Southern analysis (data not shown), then tetracycline was added to cell cultures to initiate the expression of *PTR1*-specific double-stranded RNA. The average cumulative growth with four different freshly derived clones is illustrated in Fig. 1A. In the absence of inducer these cells grew at essentially the same rate as WT cells. However, following induction of PTR1 dsRNA, growth ceased after 1 day, followed by a decline in cell numbers such that no motile parasites were visible after 4 days. Levels of PTR1 protein were diminished compared with WT in non-induced RNAi lines (Fig. 1A, inset) compared with the immunoglobulin heavy-chain-binding protein (BiP) used as a loading control. Densitometry of the PTR1 band relative to the BiP control band indicated that the non-induced RNAi line had 50% of WT protein levels; this decreased further to 9% 24 h after RNAi induction and was not detectable after 48 h. This RNAi phenotype was unstable; if the tetracycline-induction was repeated after a further 2–3 weeks in culture, cells either showed no growth defect with no knockdown of PTR1, or brief cytostasis followed by normal growth (Fig. S2). To confirm that the cidal effect of RNAi was due to loss of PTR1 activity, the effect of supplementation with H_4_B was examined on a freshly derived clone (Fig. 1B). Addition of 10 µM H_4_B to RNAi-induced cultures abolished the lethal growth phenotype.

**Fig. 2:**
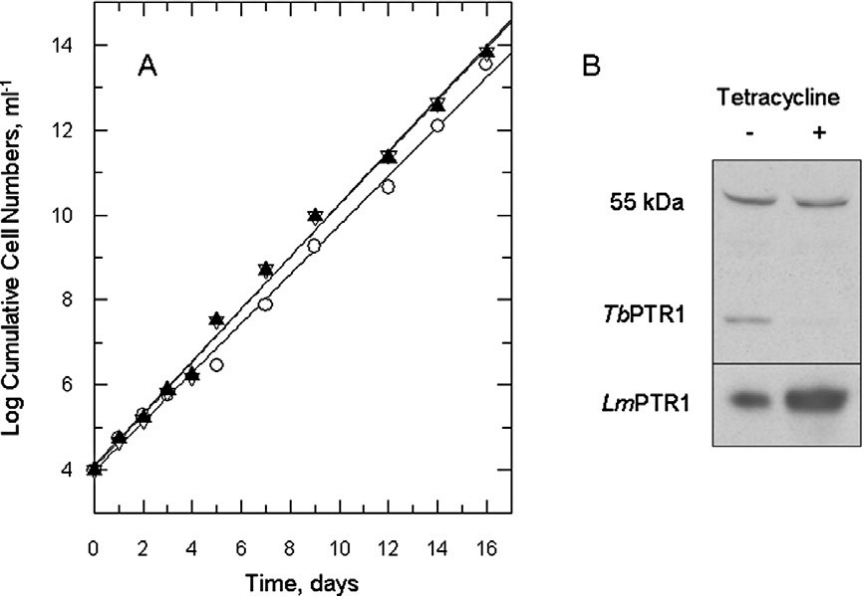
Rescue of RNAi growth defect by expression of *Lm*PTR1. A. Cumulative growth of WT (open circles) non-induced ^oe^RNAi cells (open inverted triangles) and tetracycline-induced ^oe^RNAi cells (closed triangles). B. Western blot analysis demonstrating knockdown of *Tb*PTR1 in ^oe^RNAi cells. Non-induced (-) and 48 h tetracycline induced (+) cells were probed sequentially with anti-*Tb*PTR1 and anti-*Lm*PTR1. A 55 kDa non-specific band was detected with anti-*Tb*PTR1, which was not effect by the knockdown of PTR1 and serves as an internal loading control (top panel), while *Lm*PTR1 is present in both (bottom panel).

**Fig. 3:**
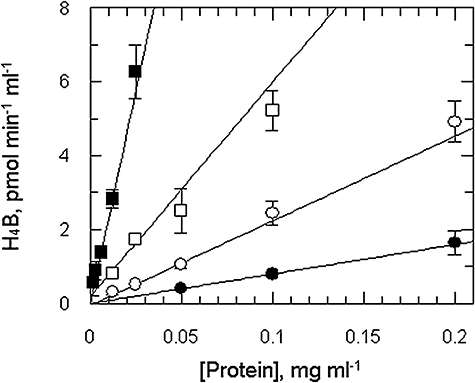
PTR1 enzyme activity in WT and transgenic *T. brucei*. Cell lysates were assayed for PTR1 activity at various protein concentrations as described in the materials and methods. WT, open circles; SKO, closed circles; ^oe^RNAi minus tetracycline, open squares; ^oe^RNAi plus tetracycline, closed squares.

Additional evidence of the essential requirement for PTR1 activity was obtained by introducing an inducible overexpressing ectopic copy of *L. major PTR1* into the WT line (^oe^WT) prior to generation of *Tb*PTR1 RNAi lines (^oe^RNAi). Due to the high GC content of the *L. major* gene, the sequence identity with the *T. brucei* RNAi construct is less than 50%, with no more than 13 nucleotide stretches of identity. Thus, *Lm*PTR1 mRNA should not be susceptible to degradation, since assembly of an RNA-induced silencing complex requires formation of 20–26-mer double-stranded RNA (Ullu *et al*., 2004). Indeed, following tetracycline induction, ^oe^RNAi cells showed no sign of any growth defect whatsoever (Fig. 2A). Western blotting with antisera raised against recombinant *T. brucei* PTR1 showed that *Tb*PTR1 expression is undetectable following induction (Fig. 2B; *Tb*PTR1 is ∼30 kDa and the 55 kDa cross-reacting band serves as an internal loading control). In contrast, Western blotting with a second antiserum specific to *L. major* PTR1 shows the opposite effect. *Lm*PTR1 is visible as a ∼30 kDa band in non-induced (due to leaky nature of the promoter in pLew82) which increases following induction. PTR1 activity was measured in cell lysates to validate these results. As shown in Fig. 3, PTR1 activity is proportional to protein concentration for each cell line. WT cells have a specific activity of 22.9 ± 1.9 nmol min mg^−1^ and this is decreased to 8.0 ± 0.2 nmol min mg^−1^ in the SKO line, whereas this increases by 2.5- and 10-fold in RNA lines overexpressing *Lm*PTR1 in the absence or presence of inducer (58.1 ± 9.3 and 231 ± 19 nmol min mg^−1^ respectively). These results demonstrate that *Lm*PTR1 is enzymatically active when expressed in *T. brucei* and is able to rescue the lethal RNAi phenotype.

**Fig. 4:**
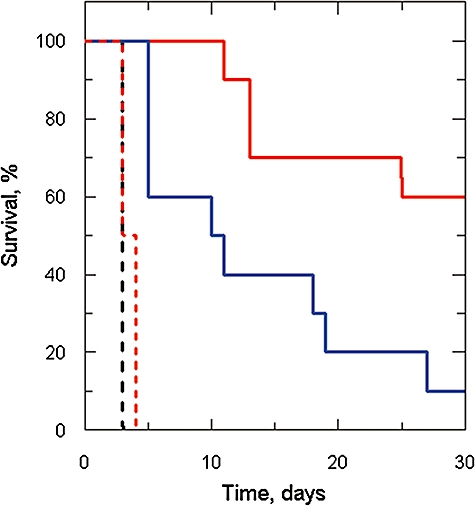
Virulence phenotype of PTR1 mutants in mice. Groups of five mice were infected with WT, non-induced cells for RNAi and ^oe^RNAi cells and parasitaemia monitored at intervals over a 30 day period. The Kaplan-Meier survival graph shows the aggregated results of two independent experiments. Symbols: WT infection, no doxycycline (black dashed line); RNAi infection, no doxycycline (blue solid line); RNAi infection plus doxycycline (red solid line); ^oe^RNAi infection, no doxycycline (red dashed line); ^oe^RNAi infection with doxycycline (black dashed line, identical to WT infection).

Infectivity studies were undertaken in mice to examine the virulence of RNAi and ^oe^RNAi transgenic trypanosomes. Wild-type and non-induced transgenic cells were inoculated into paired groups of mice, where one pair received doxycycline in their drinking water. Parasitaemia was monitored at intervals for 30 days; animals achieving a parasitaemia greater than 10^8^ trypanosomes per millilitre were euthanized as previous studies had established that these levels were consistently lethal within the next 24 h. Mice infected with 10^5^ WT cells were unable to survive longer than 3–4 days (Fig. 4), whereas mice infected with RNAi cells survived for much longer regardless of doxycycline treatment. The reduced virulence observed in the minus doxycycline group was probably due to leaky transcription of the p2T7 promoter, which lowers the baseline expression of PTR1 (Fig. 1A) and in some instances is associated with an initial lag in growth compared with WT (Fig. 1B). Six out of 10 mice treated with doxycycline failed to develop a parasitaemia and survived beyond 30 days. Infection with ^oe^RNAi cells (with or without doxycycline) restored the virulence phenotype to that of WT cells.

### Morphological changes associated with PTR1 depletion

Severe morphological abnormalities could be observed in bloodstream trypanosomes by light and electron microscopy prior to cell death. After induction of RNAi for 24–48 h, cells showed reduced motility, with some fattening of the cell body and shortening of the flagellum compared with the control (non-induced) samples. After 72 h, Giemsa-stained preparations showed gross distension of the cell body with multiple nuclei, kinetoplasts and flagella.

**Fig. 5:**
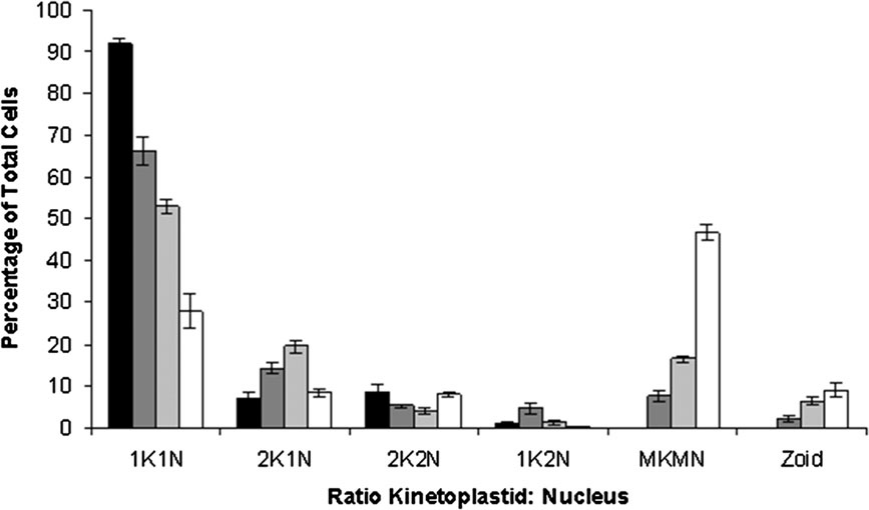
PTR1 depletion results in changes to cell cycle progression. Microscopic analysis of DAPI stained RNAi cells of non-induced (0 h) and induced (24, 48 and 72 h) populations analysed to determine the proportion of trypanosomes with different numbers of kinetoplasts (K) and nuclei (N). Percentage bar chart depicts changes in the K/N content per cell after tetracycline induction of RNAi. Approximately 300 parasites per population were analysed (*n* = 2, ± SD): non-induced control (black bars); 24 h induction (dark grey bars); 48 h induction (light grey bars) and 72 h induction (white bars).

To provide an insight into any changes in the cell cycle progression, DAPI-stained cells were analysed to determine the proportion of cells with one or more nucleus (N) and kinetoplast (K) from control, non-induced and tetracycline induced populations (Fig. 5). Non-induced samples were predominately 1K1N (92%), whereas tetracycline induction caused a dramatic time-dependent decrease in the number of 1K1N trypanosomes (28% at 72 h) with a corresponding increase in cells containing either multiple kinetoplasts plus multiple nuclei (MKMN) or anucleated zoids (1K0N). The number of cells with two kinetoplasts and one nucleus (2K1N) increased threefold from 6% to 19% by 48 h after induction, decreasing to 8% at 72 h. 2K2N did not vary markedly throughout the time course. These results suggest that most cells can undergo several rounds of mitosis, but not cytokinesis.

**Fig. 6:**
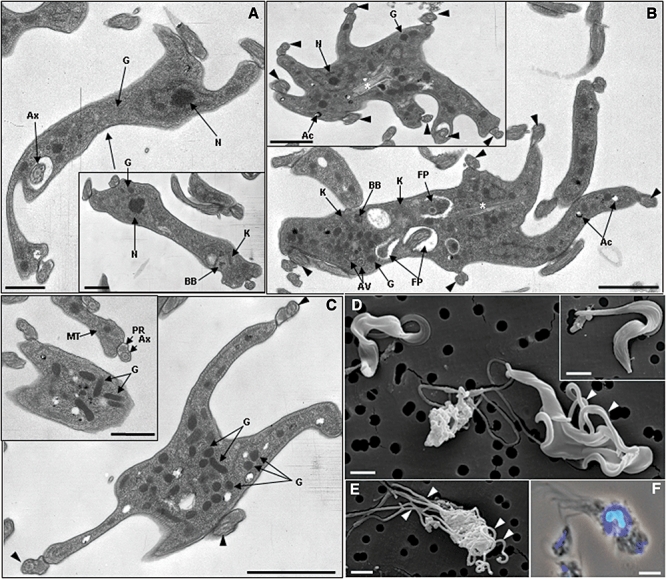
Morphology of PTR1-depleted cells by electron microscopy. A. TEM section of non-induced controls. B and C. TEM sections of 72-h induced cells depicting increased number of flagella and glycosomes. D. SEM images of 72-h induced cells, one with a normal (top left) and abnormal (bottom right) appearance compared with non-induced control (inset). E. Equivalent DAPI-stained 72-h induced cell with multiple flagella. Abbreviations used in TEM sections: nucleus (N); nuclear membrane (NM); basal bodies (BB); kinetoplast (K); flagellar rod (FR); flagellar pocket (FP); axonemes (Ax and black arrow heads); glycosomes (G); lysosomes (L); paraxial rod (PR); microtubules (MT); acidocalcisomes (Ac); autophagic vacuoles (AV) internal non-membrane bound flagellum (*). White arrow heads in SEM images highlight flagella. Black and white bars represent 200 nm.

Ultra-structural and surface alterations were examined by transmission electron microscopy (TEM) and scanning electron microscopy (SEM) (Fig. 6). The most obvious aberrant feature present in the TEM sections of the RNAi-induced cells is the increased number of membrane bound electron-dense bodies (Fig. 6B and C) compared with the non-induced control (Fig. 6A). These round, sometimes sausage-shaped (Fig. 6C, inset) bodies are morphologically similar to glycosomes, an organelle related to peroxisomes (Opperdoes *et al*., 1984). Another distinct feature in these treated cells is the increased number of flagella present in the 72 h sections (Fig. 6B–E, arrowheads). Even though there are an aberrant number of flagella associated with the main body of the parasite, they appear to have the normal 9 + 2 arrangement of the microtubules of the axoneme and paraflagellar rod, suggesting that the structural organisation of the flagellum is unaffected by PTR1 depletion. Some sections also appear to have more than one flagellar pocket (Fig. 6B), as well as a number of autophagic vacuoles. Lastly, structures were evident in some sections, which appeared to be an internal non-membrane bound flagellum (Fig. 6B plus inset, starred), a phenotype previously observed in the knockdown of VSG (Sheader *et al*., 2005). Compared with a normal trypanosome (Fig. 6D, inset), tetracycline-induced trypanosomes show an increase in body size with some having sections of flagella detached from the body and lacking the undulating membrane (Fig. 6D, arrowheads). Multiple entangled flagella can be clearly identified in the SEM and DAPI- stained image (Fig. 6E and F respectively). Phase-contrast light microscopy of wet preparations showed that these grossly abnormal parasites are still viable, with multiple flagella moving in an uncoordinated fashion with detached flagella in some instances radiating from the central body in many directions. There is also considerable disruption of the parasite surface with what appears to be membrane disorganization and deterioration. The changes highlighted on the surface are probably an indication of the latter stages of the RNAi effect with cells that are close to death, as can be seen for the central parasite in Fig. 6D.

**Fig. 7:**
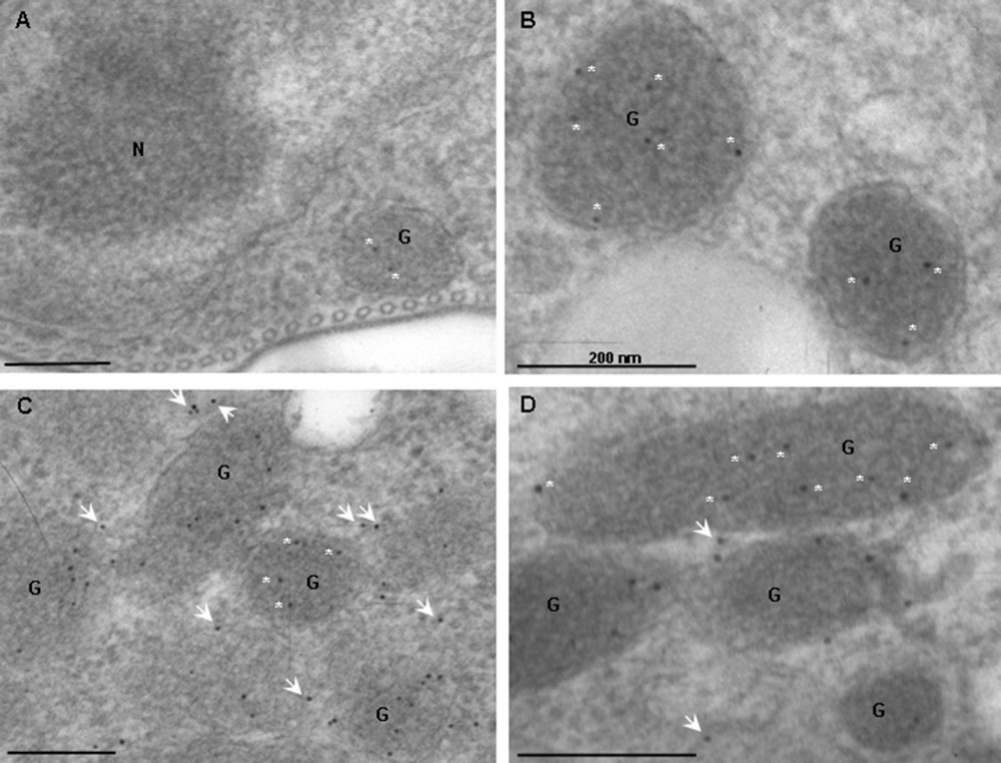
Localization and distribution of GAPDH by immuno-gold labelling. Thin-layer sections were labelled with anti-GAPDH and stained with protein A gold particles and examined by TEM. WT (A), RNAi non-induced (B) and induced (C–D) at 72 h. Abbreviations used: nucleus (N); glycosome (G) and white asterisks alongside black dots represent 10 nm gold particles present within glycosomes, while white arrows depict particles outside glycosomes. Scale bar represents 200 nm.

The identity of the electron-dense organelles as glycosomes was verified by immuno-cytochemistry. TEM sections of WT, non-induced and induced samples at 72 h were labelled with an antibody to the glycosomal matrix enzyme marker, *T. brucei* glyceraldehyde phosphate dehydrogenase (GAPDH) and visualized with 10 nm protein-A gold particles (Fig. 7). In the non-induced samples, gold particles are exclusively localized to glycosomes confirming the specificity of the antibody reagent (Fig. 7A and B). In contrast, the induced samples show pronounced labelling of glycosomes with additional gold particles in the cytosol (Fig. 7C and D, arrows). The matrix of the elongated electron-dense structure in Fig. 7D is also heavily stained confirming that the sausage-shaped structures (Fig. 6C) are likely to be glycosomes.

**Fig. 8:**
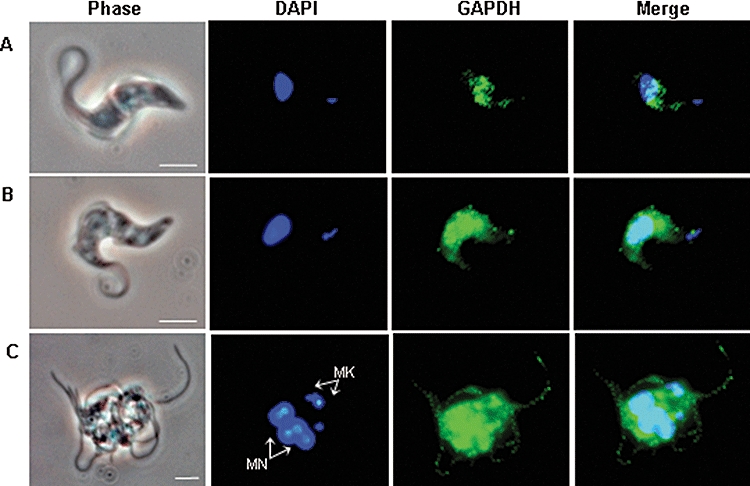
The effect of PTR1 depletion on the subcellular distribution of the glycosomal matrix enzyme, GAPDH by immunofluorescence microscopy. Non-induced (A), induced 48 h (B) and 72 h (C) RNAi cells were labelled with a glycosomal matrix marker, GAPDH (green) with staining of nuclear and kinetoplast DNA by DAPI (blue). MN, multinucleated, MK, multikinetoplastid. Scale bar represents 10 µm.

**Fig. 9:**
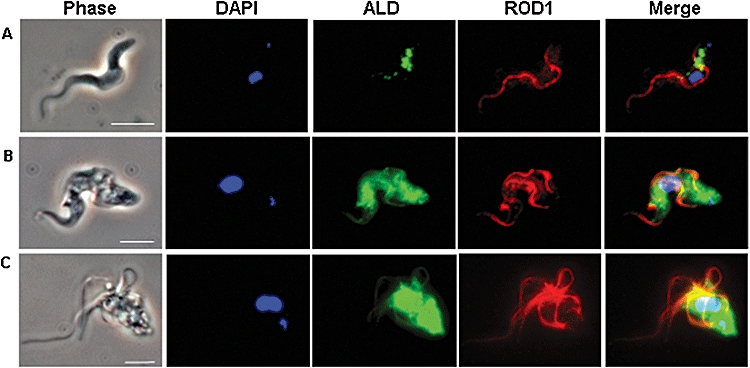
Immunolocalisation of aldolase and paraflagellar rod marker, ROD1 in RNAi depleted cells. Co-localization of aldolase (glycosomal marker, green) and ROD1 (paraflagellar rod marker, red) with DAPI (blue) on RNAi cells. Non-induced (A); induced 48 h (B); and 72 h (C) RNAi cells. MN, multinucleated, MK, multikinetoplastid, and scale bar represents 10 µm.

To verify whether there is an increase in the number of glycosomes following PTR1 depletion, immunofluorescence studies were undertaken using anti-GAPDH. Staining for GAPDH in the non-induced control is punctate in nature (Fig. 8A), which is more pronounced and diffuse following induction with tetracycline at 48 h. Staining increased to such a level that it covered almost the entire body of the parasite at 72 h. Some punctate staining is also visible along the long thin structures radiating out from the main body of these multinucleated and multikinetoplast parasites (Fig. 8C). These structures were confirmed to be detached flagella by staining with antibody to the paraflagellar rod protein, ROD1 (Fig. 9). The increase in glycosomal staining pattern illustrated with GAPDH following RNAi expression was confirmed using an alternative glycosomal matrix marker, aldolase (ALD). Induction at 48 h resulted in a partial diffuse staining into the cytoplasm; however, unlike GAPDH, no punctuate staining was visible in the flagella at 72 h.

### Subcellular distribution of PTR1 and glycosomal markers

**Fig. 10:**
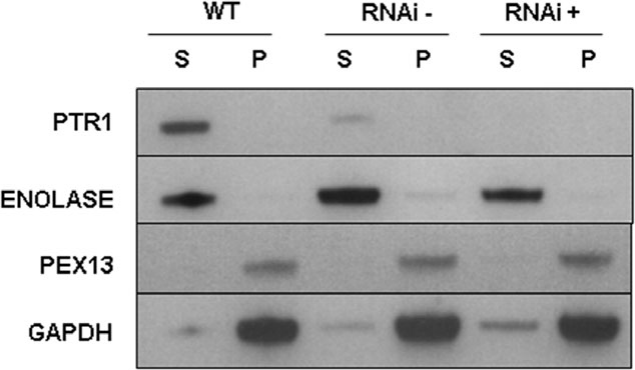
The effect of PTR1 depletion on the subcellular distribution of glycolytic enzymes. Digitonin fractionation of intact WT and RNAi cells grown for 48 h (in the absence ‘–’ and presence ‘+’ of tetracycline) were incubated with 150 µg ml^−1^ of digitonin and processed as described in Material and Methods. The release of PTR1, enolase, PEX13 and GAPDH from digitonin-treated cells was determined in supernatant (S) and pellet (P) fractions by Western blot.

Cytosolic and glycosomal fractions of bloodstream form *T. brucei* were prepared by ultracentrifugation, following digitonin treatment of WT and 48 h induced RNAi cells and analysed by Western blotting with antibodies to specific cytosolic and glycosomal markers (Fig. 10). PTR1 was recovered exclusively in the cytosolic fraction along with the cytosolic enzyme marker, enolase. Similar results were obtained for procyclic forms (Fig. S3). Reduced levels of PTR1 are evident in the cytosolic non-induced fraction of RNAi cells compared with WT, due to the leaky nature of p2T7^Ti^TAblue RNAi vector (Alibu *et al*., 2005). Following RNAi induction for 48 h, PTR1 is below the limit of detection, whereas enolase and PEX13, a glycosomal membrane-bound protein, were unchanged. Densitometry of the band stained for the glycosomal matrix enzyme GAPDH indicated that the pellet fraction was unchanged whereas the amount in the cytosol was increased approximately twofold. Although analysis of 72 h induced samples was not possible due to lack of material, the increased amount of GAPDH observed 48 h after induction, supports the mis-localization of GAPDH to the cytosol illustrated in the immuno-gold labelling studies at 72 h (Fig. 7).

## Discussion

The gene knockout, RNAi knockdown and rescue studies reported here provide strong evidence that PTR1 is essential for the blood-stream form of the African trypanosome. Loss of PTR1 by RNAi is cytocidal rather than cytostatic, which is a distinct advantage from a drug discovery perspective. This lethal RNAi phenotype can be rescued by expression of functionally active *L. major PTR1*, or by supplementation with tetrahydrobiopterin. PTR1 levels were markedly reduced even in the absence of inducer due to poor regulation of the pLew82 vector. While this had no discernable effect on growth in rich medium *in vitro*, the virulence of the PTR1 RNAi *T. brucei* was considerably attenuated in mice. Further loss of virulence was evident when RNAi was induced, but some mice still succumbed to infection. This is most likely due to the instability of the RNAi machinery as previously reported (Chen *et al*., 2003). However, since supplementation with 10 µM tetrahydrobiopterin was able to rescue the trypanocidal effect *in vitro*, we cannot definitively exclude the possibility that the steady-state levels of tetrahydrobiopterin present within the animal host plasma (∼0.1 µM) are contributing to survival (Fukushima and Nixon, 1980; Yoshida *et al*., 2000). The fact that BH_4_ is extremely unstable in culture medium and that repeated daily addition of BH_4_ was found to be toxic to *T. brucei* could explain the failure to generate a double knockout line using BH_4_ as a nutritional rescue. Thus, ultimate proof that PTR1 is a suitable drug target on its own requires chemical validation using potent and specific drug-like inhibitors of PTR1 with appropriate pharmacological properties. Unfortunately, the currently available inhibitors do not meet these specifications (Cavazzuti *et al*., 2008; Mpamhanga *et al*., 2009; Shanks *et al*., 2010). Nonetheless, combined inhibition of dihydrofolate reductase and PTR1 remains an attractive chemotherapeutic strategy (Hardy *et al*., 1997; Cavazzuti *et al*., 2008; Tulloch *et al*., 2010).

Our morphological data illustrate that knockdown of PTR1 results in cells that are unable to divide normally, although they can still undergo nuclear division and kinetoplast segregation. Formation of multiflagellated parasites has also been reported in a number of other RNAi studies dissecting organelle biogenesis including: depletion of trypanin, a component of the dynein regulatory complex of the flagellum (Ralston and Hill, 2006); depletion of centrin1, a component associated with the basal bodies and responsible for segregation of the linked organelles: flagellum, kinetoplasts and Golgi (Selvapandiyan *et al*., 2007); depletion of the aurora-like kinase AUK1, a chromosome passenger protein (Li and Wang, 2006; Jetton *et al*., 2009); and depletion of CAP5.5 resulting in defects in microtubule organization (Olego-Fernandez *et al*., 2009). However, none of these studies have observed the glycosomal abnormalities reported here. Further work is required to determine whether other RNAi mutants with cytokinesis defects might show similar glycosomal abnormalities.

Knockdown of PTR1 also affects glycosomal numbers and their morphology, as judged by TEM. PTR1 knockdown also causes partial mislocalisation to the cytosol of the glycosomal matrix enzymes, GAPDH and aldolase, as seen in the immunofluorescence and digitonin fractionation experiments. In our view, mislocalization could be due to either defective import into the glycosome or due to loss of integrity of the glycosomal membrane. However, similar effects observed in studies on the functional roles of peroxins have been attributed to defective import. Peroxins (PEX) are a large family of peroxisomal proteins which are involved in glycosomal biogenesis via the import of newly synthesized proteins from the cytosol (Parsons *et al*., 2001). Cellular knockdown by RNA interference of PEX 13 (Furuya *et al*., 2002; Verplaetse *et al*., 2009), GIM5 (a PEX11-related protein) (Furuya *et al*., 2002; Moyersoen *et al*., 2003; Voncken *et al*., 2003; Verplaetse *et al*., 2009), PEX14 (Moyersoen *et al*., 2003), PEX19 (Banerjee *et al*., 2005); and PEX6, PEX10 and PEX12 (Krazy and Michels, 2006) all resulted in the partial mislocalization of glycosomal matrix enzymes to the cytosol. Overexpression of PEX11 led to an increased number of glycosomes which were elongated and clustered (Lorenz *et al*., 1998), whereas knockdown of PEX19 resulted in smaller numbers of larger glycosomes (Banerjee *et al*., 2005). However, none of these studies reported defects in cytokinesis that we observed here.

The discovery that *Tb*PTR1 is present within the cytosol in bloodstream (Fig. 10) and procyclic forms (Fig. S3) refutes the predicted glycosomal location for PTR1, based on the PST1 consensus sequence -VHA at its C-terminus (Opperdoes and Szikora, 2006). This is consistent with the failure to detect PTR1 by a proteomic analysis of purified *T. brucei* glycosomes (Vertommen *et al*., 2008) and agrees with the cytosolic location of a GFP-PTR1 chimera in *L. donovani* (Kumar *et al*., 2007). Likewise, both trypanothione reductase and trypanothione peroxidase from *T. brucei* also contain putative glycosomal targeting sequences, yet the former is clearly cytosolic (Smith *et al*., 1991) and the latter is located in the cytosol and mitochondria (Schlecker *et al*., 2005). This underlines the need for experimental verification of any in silico bioinformatic predictions (Wanders and Waterham, 2006).

PTR1 clearly has pleiotropic effects on cytokinesis, flagellar morphology and glycosome biogenesis. The challenging question is what is the underlying biochemical mechanism? Of the two known functions for PTR1, the maintenance of biopterin in its tetrahydro-form seems more likely than the salvage of folate to dihydrofolate, since the growth phenotype can be rescued by supplementation with H_4_B in high- or low-folate media containing thymidine. Since the functions of pterins remain to be elucidated in *T. brucei* one can only speculate what these functions might be. For example, part of the ether lipid biosynthetic pathway is present in mammalian peroxisomes cells (Kaufman *et al*., 1990) and in trypanosomatid glycosomes (Lux *et al*., 2000; Parsons *et al*., 2001; Michels *et al*., 2006). To date, the first three enzymes of this pathway have been identified in trypanosomatids by fractionation, bioinformatic and proteomic based analysis (Heise and Opperdoes, 1997; Opperdoes and Szikora, 2006; Vertommen *et al*., 2008), but none of these are known to be pterin-dependent. However, BH_4_ is involved in ether lipid catabolism in mammalian cells, where it serves as an essential cofactor for the cleavage of ether lipids via glyceryl ether monooxygenase (EC 1.14.16.5) located in the microsomal compartment (Watschinger *et al*., 2009). The ultimate products of ether lipid cleavage are glycerol and a fatty alcohol. Possibly, these fatty alcohols could serve in the subsequent ether lipid synthesis in the glycosome. However, the fact that oxidative cleavage of ether lipids in *L. donovani* was found to be dependent on NADPH rather than tetrahydrobiopterin (Ma *et al*., 1996) tends to argue against this hypothesis. Tetrahydrobiopterin also plays a role in defence against oxidative and nitrosative stress in mammalian cells (Mogi *et al*., 1999; Ozkor and Quyyumi, 2008) and recent studies in PTR1 knockout and overexpressing lines suggest a similar role for *Leishmania spp.* (Moreira *et al*., 2009; Nare *et al*., 2009).

In conclusion, our data strongly suggest that PTR1 is essential for blood stream *T. brucei* for growth and survival *in vitro* and in the animal host. The phenotypic abnormalities uncovered in this investigation suggest a potential role for PTR1 in the biogenesis of the glycosome and the flagellum. Further studies are underway in our laboratory to investigate these and other possibilities.

## Experimental procedures

### Trypanosome culture

*Trypanosoma brucei* bloodstream form ‘single marker’ S427 (*T7RPOL TETR NEO*) and transfected cells were cultured as previously described (Wirtz *et al*., 1999) and (Alibu *et al*., 2005). Folate deficient media (FDM) and growth conditions used in the study were as previously described (Sienkiewicz *et al*., 2008). Tetrahydrobiopterin was purchased from B. Shircks Laboratories (Jona, Switzerland) and 10 mM stock was prepared in degassed 0.1 M HCl.

### Cloning and generation of transgenic cell lines

All constructs made were sequenced and prepared for electroporation using QIAprep Miniprep Plasmid Kit (Qiagen). DNA constructs were prepared for electroporation into trypanosomes and cloned with all procedures performed as previously described (Wirtz *et al*., 1999; Sienkiewicz *et al*., 2008). Primers used in this study to generate the RNAi and overexpressor constructs (Table S1) are based on the GeneDB sequences for *T. brucei PTR1* (Tb927.8.2210, 927 strain) and *L. major PTR1* (LmjF23.0270, Friedlin strain). For the disruption of PTR1 function, a 403 bp region (nucleotide position 399–801; http://trypanofan.path.cam.ac.uk/software/RNAit.html) was directly ligated into the p2T7^Ti^TAblue vector (http://trypanofan.path.cam.ac.uk/trypanofan/vector/; a generous gift from David Horn), linearized by digestion with NotI and transfected by electroporation to generate RNAi cells under hygromycin selection (4 µg ml^−1^). An overexpressor construct (pLew82), containing the *L. major PTR1* ORF was also made for generation of an overexpressor WT (^oe^WT) under phleomycin selection (2.5 µg ml^−1^). The ^oe^WT cells generated were used for the reintroduction of the p2T7^Ti^TAblue RNAi construct to generate ^oe^RNAi cells. All tetracycline induction experiments were initiated at 1 × 10^4^ cells ml^−1^ and tetracycline added daily at 1 µg ml^−1^.

### DNA analysis

The WT and RNAi genomic DNA (5 µg) was digested with the restriction enzyme StuI and blots were subsequently probed with *TbPTR1* ORF as described previously (Sienkiewicz *et al*., 2008). Southern blot analysis was used to confirm the successful integration of the selectable gene markers into WT cells (see *Supporting information*).

### Western blot analysis

The WT, RNAi (0, 24 and 48 h) and ^oe^RNAi (48 h) cells were centrifuged at 20 000 g for 5 min at 4°C. The cell pellets (1 × 10^6^ parasites per sample) were resuspended in SDS Laemmli buffer, boiled for 5 min and micro-centrifuged as above. The supernatants were then separated by SDS-PAGE and subsequently transferred onto nitrocellulose for immunoblotting as previously described (Sienkiewicz *et al*., 2008). The immunoblot for the RNAi set was sequentially probed with polyclonal antisera raised against *T. brucei* PTR1 (1:500 dilution) and BiP (1:10 000), in conjunction with a secondary rabbit anti-rat HRP-conjugated antibody (1:5 000, Dako). The ^oe^RNAi samples were sequentially probed with *T. brucei* PTR1 antisera and *L. major* PTR1 (1:10 000), in conjunction with secondary rabbit anti-rat and goat anti-rabbit HRP-conjugated antibodies (1:5000 and 1:10 000 respectively) and the immunoblots visualized with the ECL plus (enhanced chemiluminescence) system from GE Healthcare.

### Enzyme assays

Lysates of *T. brucei* were prepared as previously described (Vickers and Fairlamb, 2004). To ensure adequate extraction of parasites, trypanothione reductase activity was measured in clarified lysates as previously described (Jockers-Scherubl *et al*., 1989). Assays for PTR1 activity in clarified lysates were carried out at 22°C in 50 mM HEPES (pH 7.4), containing 0.1% (v/v) triton X-100, adjusted to an ionic strength of 100 mM with KCl. Lysates were preincubated with 100 µM NADPH cofactor for 2 min and reactions were initiated by the addition of 25 nM H_2_B, in a final volume of 200 µl. Enzymatic reactions were further incubated for 1 min, before aliquots (100 µl) were removed and oxidized with iodine under alkaline conditions and analysed by HPLC as previously described (Shanks *et al*., 2010). Protein was measured using Coomassie blue using bovine serum albumin as standard (Bradford, 1976).

### Morphological analysis

RNAi cells (grown in the presence and absence of tetracycline) were fixed with methanol or 4% (v/v) paraformaldehyde and stained with either Giemsa or 4′,6-diamidino-2-phenylindole (Slowfade Gold antifade reagent with DAPI, Invitrogen). Images were captured on a Zeiss Axiovert 200 M fluorescence and light microscope using Zeiss AxioVision Software. Approximately 300 parasites per population were assessed for changes in N:K ratio and data presented as the mean of two independent knockdown experiments. For electron microscopy, cells were fixed for 24 h in 2% (v/v) glutaraldehyde and 4% (v/v) paraformaldehyde in 0.2 M PIPES, pH 7.2 and prepared for both SEM and TEM as previously described (Sienkiewicz *et al*., 2008).

### Immuno-gold labelling of TEM sections

For immunoelectron microscopy the labelling and staining procedure was carried out as essentially described (Sandilands *et al*., 2004; Sienkiewicz *et al*., 2008). Briefly, samples previously embedded in Durcupan resin were etched with 1% (v/v) periodic acid and the osmium removed by 2% (w/v) sodium periodate. The sections were then probed with anti-GAPDH (1: 50 dilution) antibody and subsequently incubated with Protein A labelled with 10 nm gold particles (1:50 dilution, BB International) to visualize the primary antibody. The sections were stained with 3% (v/v) aqueous uranyl acetate and Reynold's lead citrate prior to examination with a JEOl-1200 EX TEM.

### Immunofluorescence

Cells were fixed with 4% (v/v) paraformaldehyde onto poly-L-lysine coated chamber slides and probed with antisera raised against *T. brucei* GAPDH (rabbit antiserum, 1:2500), ALD (rabbit antiserum, 1:6000) and ROD1 (monoclonal antiserum, neat), generous gifts from Paul Michels and Keith Gull, as previously described (Woods *et al*., 1989; Verplaetse *et al*., 2009). Combination of secondary Alexafluor 488 and 555 conjugated ant-rabbit or anti-mouse immunoglobulins were used (both 1:800 dilution, Molecular Probes) and slides were counter-stained with DAPI prior to analysis. Images were captured using FITC, Texas Red and DAPI filters as previously described. Property parameters were set on the AxioVision microscope software to optimize the intensity of the fluorescent stains standardized against the non-induced controls and these values were retained for all images captured. Images shown are representative of three independent experiments.

### Separation of cytosolic and glycosomal rich proteins by digitonin permeabilization

Digitonin permeabilization experiments were undertaken to separate cytosolic and glycosomal rich proteins from WT and RNAi cells (grown in the absence and presence of tetracycline for 48 h), as previously described (Voncken *et al*., 2003). Briefly, live cells were washed once in STEN (250 mM sucrose, 25 mM Tri-HCl, 1 mM EDTA, 150 mM NaCl, pH 7.4), pelleted and resuspended in STEN containing 150 µg ml^−1^ digitonin (at a final concentration 5 × 10^8^ cells ml^−1^) which was subsequently incubated for 1 h on ice. The digitonin treated cells were then fractionated by centrifugation (2 min, 20 000 g, at 4°C) resulting in an enriched cytosolic (s, supernatant) and glycosomal (p, pellet) fractions. Approximately 5 × 10^6^ fractionated parasites were processed for Western blot analysis and sequentially probed with the following antibodies raised against *T. brucei* PTR1 (1:500), rabbit antisera raised against PEX13 (1:20 000), enolase (1:100 000) and GAPDH (1:100 000). Blots were subsequently incubated with secondary anti-rat (1:5000 dilution) and anti-rabbit HRP-conjugated antiserum (1:15 000) and visualized as previously described.

### *In vivo* studies

All cell lines (WT, RNAi, *^oe^*RNAi) were cultured for 24 h in the absence of selectable drugs before female National Medical Research Institute (NMRI) mice (5 per group) were infected with a single intraperitoneal injection of 10^5^ parasites in 0.2 ml glucose saline as previously described (Sienkiewicz *et al*., 2008). For the RNAi and ^oe^RNAi cells, a single inoculum of non-induced cells were infected into two sets of mice with one group being given 2.5 µg ml^−1^ doxycycline in their drinking water (starting a week prior to infection and continuing throughout the 30 day period). Animals were fed *ad libitum* on standard chow (Diet 3, SAS, Edinburgh). Parasitaemia levels were monitored throughout the 30 day experiment (Sienkiewicz *et al*., 2008). All animal experiments undertaken were carried out following local ethical review and under UK regulatory licensing in accordance with the European Communities Council Directive (86/609/EEC).
